# Alpha-Galactosidase *A* p.A143T, a non-Fabry disease-causing variant

**DOI:** 10.1186/s13023-016-0441-z

**Published:** 2016-05-04

**Authors:** Malte Lenders, Frank Weidemann, Christine Kurschat, Sima Canaan-Kühl, Thomas Duning, Jörg Stypmann, Boris Schmitz, Stefanie Reiermann, Johannes Krämer, Daniela Blaschke, Christoph Wanner, Stefan-Martin Brand, Eva Brand

**Affiliations:** Internal Medicine D, Department of Nephrology, Hypertension and Rheumatology, University Hospital Muenster, Albert-Schweitzer-Campus 1, D-48149 Muenster, Germany; Department of Medicine, Divisions of Cardiology and Nephrology, Comprehensive Heart Failure Center, Fabry Center for Interdisciplinary Therapy (FAZIT), University of Wuerzburg, Wuerzburg, Germany; Katharinen-Hospital Unna, Unna, Germany; Department II of Internal Medicine and Center for Molecular Medicine Cologne, University of Cologne, Cologne, Germany; Department of Medicine, Division of Nephrology, University Hospital Charité, Campus Virchow-Klinikum, Berlin, Germany; Department of Neurology, University Hospital Muenster, Muenster, Germany; Department of Cardiovascular Medicine, Division of Cardiology, University Hospital Muenster, Muenster, Germany; Institute of Sports Medicine, Molecular Genetics of Cardiovascular Disease, University Hospital Muenster, Muenster, Germany; Department of Pediatrics an Adolescence Medicine, University of Ulm, Ulm, Germany; Department of Medicine, Division of Cardiology, University Hospital Charité, Campus Virchow-Klinikum, Berlin, Germany

**Keywords:** Fabry disease, Lyso-Gb3, Variant of unknown significance, Late-onset, GLA mutation, Stroke, Genotype

## Abstract

**Background:**

Fabry disease (FD) is an X-linked multisystemic disorder with a heterogeneous phenotype. Especially atypical or late-onset type 2 phenotypes present a therapeutical dilemma.

**Methods:**

To determine the clinical impact of the alpha-Galactosidase A (GLA) p.A143T/ c.427G > A variation, we retrospectively analyzed 25 p.A143T patients in comparison to 58 FD patients with other missense mutations.

**Results:**

p.A143T patients suffering from stroke/ transient ischemic attacks had slightly decreased residual GLA activities, and/or increased lyso-Gb3 levels, suspecting FD. However, most male p.A143T patients presented with significant residual GLA activity (~50 % of reference), which was associated with normal lyso-Gb3 levels. Additionally, p.A143T patients showed less severe FD-typical symptoms and absent FD-typical renal and cardiac involvement in comparison to FD patients with other missense mutations. Two tested female p.A143T patients with stroke/TIA did not show skewed X chromosome inactivation. No accumulation of neurologic events in family members of p.A143T patients with stroke/transient ischemic attacks was observed.

**Conclusions:**

We conclude that GLA p.A143T seems to be most likely a neutral variant or a possible modifier instead of a disease-causing mutation. Therefore, we suggest that p.A143T patients with stroke/transient ischemic attacks of unknown etiology should be further evaluated, since the diagnosis of FD is not probable and subsequent ERT or chaperone treatment should not be an unreflected option.

**Electronic supplementary material:**

The online version of this article (doi:10.1186/s13023-016-0441-z) contains supplementary material, which is available to authorized users.

## Background

Fabry disease (FD; OMIM #301500) is an X-linked (Xq22.1) inborn error of glycosphingolipid catabolism due to deficient α-galactosidase A activity (GLA; 300644; [EC 3.2.1.22]). *GLA* mutations may lead to a classical or non-classical FD phenotype [1]. A clear link between genotype and phenotype in FD has not yet been established. Classical FD manifestations lead to clinical manifestations such as early stroke, malignant cardiac arrhythmia, myocardial infarction, cardiac failure, left ventricular hypertrophy (LVH) as well as progressive renal impairment, associated with differential systemic cellular accumulation of globotriaoslyceramide (Gb3). Several missense and nonsense mutations are accompanied by nearly absent GLA activities and increased lyso-Gb3 levels [2, 3]. Most non-classical FD phenotypes result either from missense mutations or from mutations within intronic or regulatory *GLA* regions, lacking classical FD symptoms. Affected patients suffer from late-onset organ manifestations such as cardiomyopathy, kidney failure, stroke, or neuropathic pain accompanied by high residual GLA activities and lyso-Gb3 values within the reference values, as recently shown for several non-classical FD mutations [4–12]. Due to the high frequencies of private mutations in FD patients, most genotype-phenotype relationships of controversially discussed mutations have been based on single case studies, necessitating multicenter approaches with larger patient groups carrying identical mutations. This approach will also allow for the assessment of whether affected patients may benefit from enzyme replacement therapy (ERT).

Recent studies considered the c.427G > A (p.A143T) *GLA* mutation to be more likely a neutral variant or a possible modifier instead of a disease causing mutation [10, 13]. In the current work, we retrospectively analyzed the first visit of 25 ERT naïve adult female and male patients, carrying the controversially discussed p.A143T variation in a multicenter study of four German FD centers and compared these data gender-specific to 58 ERT naïve patients (39 females) with other missense *GLA* mutations to determine a potential clinical impact of p.A143T.

## Methods

### Study design and patients

Between July 2006 and March 2014 twenty-five genetically confirmed adult heterozygous (females) and hemizygous (males) FD patients with the p.A143T mutation were consecutively recruited at Fabry centers of the University hospitals in Muenster, Wuerzburg, Cologne, and the Charité Berlin. Patients were retrospectively analyzed in an open cohort study and compared gender-specifically to 58 FD patients with other missense mutations (*n* = 24). All patients including patients with p.A143T within this study were either heterozygous (females) or hemizygous (males) carriers for the corresponding *GLA* mutations. Missense mutations included single nucleotide substitutions within the *GLA* coding region, resulting in a single substitution of amino acids, generally associated with a milder FD phenotype, compared to patients with nonsense mutations. A detailed overview of all analyzed mutations is provided in the supplement (Additional file 1: Table S1). All investigations were performed after approval of the Ethical Committees of the participating centers (Medical Association of Westfalian-Lippe and the Ethical Committee of the Medical Faculty of the University of Muenster; Ethical Committee of the Medical Faculty of the University of Cologne; Ethical Committee of the Medical Faculty of the University of Wuerzburg; Ethical Committee of the Charite Berlin, project-no: 2011–347-f; 14–328) and written informed consent of the patients for molecular analysis and publication was obtained.

A comprehensive diagnostic work-up had been performed in all centers, including medical history and cardiac, renal, and neurologic evaluation. The documentation of assessments follows the clinical practice of the German Fabry Expert Centers for a rare multisystemic disorder [14]. Cardiac assessment included echocardiography and electrocardiography. LVH was defined as an interventricular septum thickness in diastole (IVSd) >12 mm. Renal function was quantified by the estimated glomerular filtration rate (eGFR) using the Chronic Kidney Disease-Epidemiology Collaboration equation (CKD-EPI [15]) and the albumin-to-creatinine ratio (ACR) from spot urine. Renal impairment was defined as eGFR <90 ml/min/1.73 m^2^ according to KDIGO guidelines [16] and albuminuria as ACR >30 mg albumin per g creatinine. All patients underwent neurologic examination and a clinical interview focusing on a history of stroke or transient ischemic attack (TIA), and neuropathic pain. Strokes/TIAs were classified according to Trial of ORG 10172 in Acute Stroke Treatment (TOAST) criteria [17]. Disease severity scores were assessed using the Mainz Severity Score Index (MSSI [18]) and the total Disease Severity Scoring System (DS3 [19]).

### GLA sequencing, measurement of GLA activity and plasma lyso-Gb3

Genotyping for *GLA* gene mutations was performed by direct sequencing of all 7 coding exons including adjacent intron-exons boundaries as previously reported [20]. GLA activity was determined using 4-methylumbelliferyl-α-D-galactopyranoside (Santa Cruz Biotechnology, Heidelberg, Germany) as previously described [21]. N-acetylgalactosamine (Santa Cruz Biotechnology) was used as specific inhibitor of endogenous α-Galactosidase B activity [22]. GLA enzyme activity was determined as nanomoles (nmol) of substrate hydrolyzed per hour (h) per mg protein. For plasma lyso-Gb3, lyso­Ceramide was used as reference (Matreya LLC, Pleasant Gap, USA) and D5­Fluticasone Propionate (EJY Tech, Inc., Rockville, USA) served as internal standard as described elsewhere. Plasma lyso-Gb3 levels were measured in the lab of Arndt Rolfs (University of Rostock, Germany). Reference values for lyso-Gb3 were <0.9 ng/ml in plasma and for GLA activity >32 nmol/h/mg protein in leukocytes. ERT-naïve lyso-Gb3 values were available for 10 male and 14 female p.A143T patients and 14 male and 26 female patients with missense mutations. X chromosome inactivation analysis was performed with genomic DNA from leukocytes using a modified human androgen receptor (HUMARA) assay. In short, 150 ng DNA were either incubated for 12 h at 37 °C with 4 units *Rsa*I, or *Rsa*I with *Hpa*II (all New England Biolabs, Frankfurt am Main, Germany). Thirty ng DNA were used as template for subsequent amplification with KAPAHifi (Peqlab, Erlangen, Germany) using the primer combination HUM_fw: 5’-GCGCGAAGTGATCCAG-3’ and HUM_rev: 5’GCCTCTACGATGGGCTTG-3’. Amplicons were separated on a 3.5 % agarosegel and stained with ethidium bromide. Subsequent analysis according to Allen and colleagues [23] was performed with ImageJ (NIH, Bethesda, USA; http://imagej.nih.gov/ij/).

### Statistical analysis

Eighty-three patients from the participating FD centers were included in the analysis. If not stated otherwise, continuous variables were expressed as mean with standard deviation, or in case of unequal distribution as median [range]. Categorical data were expressed as numbers and relative frequencies in percent. Differences between groups were analyzed with the unpaired Student’s t or Mann–Whitney U test for continuous data, and the Fisher’s exact test for categorical data. Statistical significance was considered at a 2-sided *p* < 0.05.

All results are reported with their respective 95 % confidence intervals (CI). SAS version 9.3 (SAS Institute Inc, Cary, USA) and GraphPad PRISM V5.0 software (GraphPad Software Inc., La Jolla, USA) were used for all statistical analyses.

## Results

**Fig. 1:**
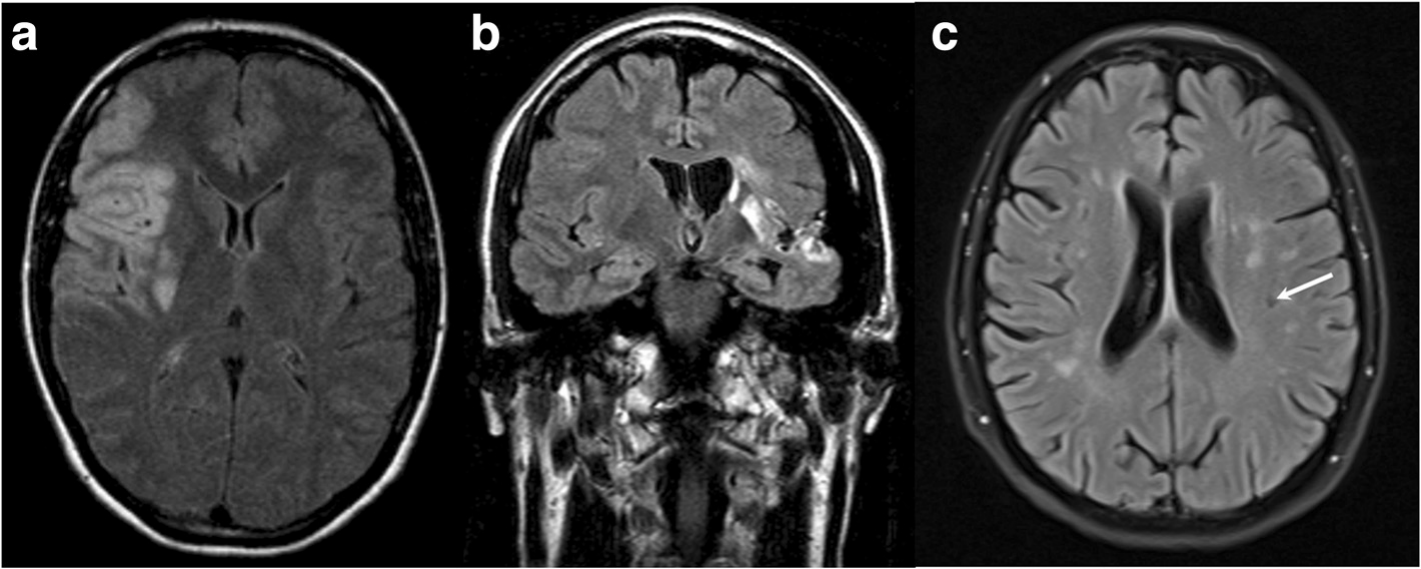
Magnetic resonance (MR) images of p.A143T patients with cerebrovascular events. **a** Axial MRI of the index female patient (30 years of age) suffering from an embolic stroke within the vascular territory of the right middle cerebral artery. **b** Coronal FLAIR-MRI of a male patient (38 years of age) suffering from an embolic stroke within the vascular territory of the left middle cerebral artery. **c** FLAIR-MR of a female patient (46 years of age) with TIAs and periventricular and subcortical microangiopathic and lacunar ischemic lesions (white arrow). FLAIR: fluid attenuated inversion recovery. TIA: transient ischemic attack

An index patient for the current study was a female, 30 years of age, presenting at the IFAZ Muenster in 2012 after a stroke (media infarction) of unknown etiology (Fig. 1a). The patient revealed an unrevealing cardiovascular risk profile and no evidence of extracerebral arteriopathy, vasculitis or other inflammatory disease. Direct *GLA* sequencing identified the p.A143T (c.427G > A, SNP# rs10489845) mutation, but plasma lyso-Gb3 level was normal. A family screening identified her father (age: 64 years), her uncle (father’s brother; age: 63 years) with his daughter (age: 32 years) also with the p.A143T variant. Although both males showed a slightly reduced GLA activity of ~50 % of the reference level none of the three relatives presented with increased plasma lyso-Gb3 levels, nor other FD-typical organ manifestations. The family history revealed strokes of her paternal grandparents at a higher age, but not in any further relatives of the index patient (neither in p.A143T affected, nor unaffected). Since at this time-point no other stroke-causing diseases could be detected within the index patient, FD was diagnosed and ERT with agalsidase-alfa was initiated, although the patient showed no further FD-typical renal or cardiac manifestations.

**Fig. 2:**
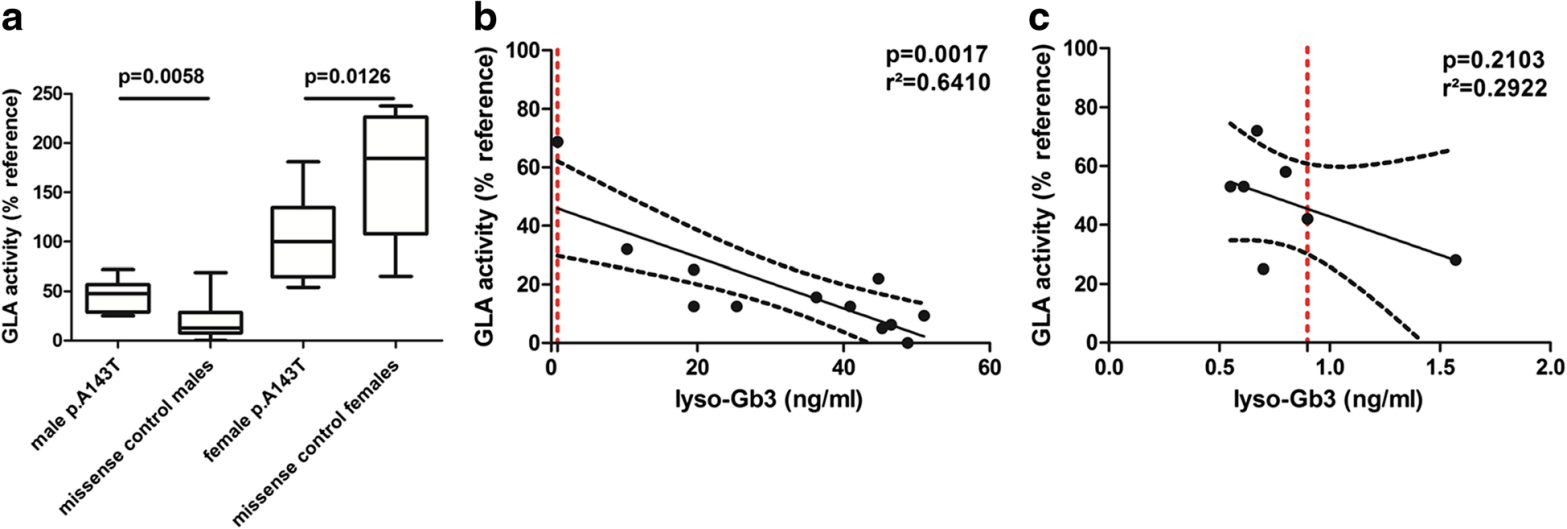
**a** Distribution of residual GLA activities in patients with p.A143T and Fabry disease patients with other missense *GLA* mutations. **b** Correlation of residual GLA activities with lyso-Gb3 levels in male Fabry disease patients with other missense *GLA* mutations and **c** in male patients with p.A143T. The dotted line represents the upper reference value of measured lyso-Gb3 (0.9 ng/ml). Values are given as medians with 5–95 % confidence intervals

Since the effect of the GLA p.A143T variation is discussed controversially and it has been recently described as a non-pathogenic *GLA* variant instead of a FD-causing mutation [10, 13], we retrospectively analyzed each first visit between July 2006 and March 2014 of p.A143T patients, to determine its potential clinical impact. Twenty-one additional patients of another 11 unrelated families with the p.A143T mutation were consecutively recruited at Fabry centers in Muenster, Wuerzburg, Cologne and Berlin. A summary of clinical symptoms and manifestations in patients with p.A143T and other missense mutations is provided in Table 1. Even if females with p.A143T suffered more often from unspecific GI symptoms such as abdominal pain (*p* = 0.0133) and diarrhea (*p* = 0.0048), all other analyzed parameters did not differ (Table 1). For subsequent analyses, naïve p.A143T patients were gender-specifically compared to naïve FD patients with other *GLA* missense mutations concerning organ manifestations and clinical events (Table 2). A detailed overview of the analyzed *GLA* mutations is provided in the supplement (Additional file 1: Table S1). GLA activity in all tested hemizygous males with p.A143T was below the reference, but significantly higher compared to males with other missense mutations (Table 2; Fig. 2a). GLA activity values in heterozygous females with the p.A143T variant were lower compared to heterozygous females with other missense mutations (*p* < 0.05), but still above reference (Table 2; Fig. 2a). In contrast to previous studies [13] no tissue biopsies have been performed.

**Table 1:** Symptoms and manifestations in p.A143T patients and patients with missense *GLA* mutations

	p.A143T Fabry patients			Missense mutation patients		
	Total (*n* = 25)	Female (*n* = 15)	Male (*n* = 10)	Total (*n* = 58)	Female (*n* = 39)	Male (*n* = 19)
gastrointestinal pain, n	12 (48.0)	8 (53.3)*	4 (40.0)	11 (20.0)	6 (16.2)	5 (27.8)
diarrhea, n	8 (32.0)	6 (40.0)**	2 (20.0)	5 (9.1)	2 (5.4)	3 (16.7)
tinnitus, n	7 (29.2)	4 (28.6)	3 (30.0)	6 (10.7)	6 (16.2)	0 (0.0)
neuropathic pain, n	7 (28.0)	4 (26.7)	3 (30.0)	26 (45.6)	18 (47.4)	8 (42.1)
hypohidrosis, n	6 (24.0)	4 (26.7)	2 (20.0)	18 (32.1)	11 (29.0)	7 (38.9)
fatigue, n	5 (20.0)	4 (26.7)	1 (10.0)	10 (18.9)	8 (23.5)	2 (10.5)
hypacusis, n	3 (12.5)	1 (7.1)	2 (20.0)	5 (8.8)	5 (13.2)	0 (0.0)
dyspnea, n	3 (12.0)	3 (20.0)	0 (0.0)	12 (21.1)	8 (21.1)	4 (21.1)
edema, n	1 (4.2)	1 (6.7)	0 (0.0)	2 (3.6)	0 (0.0)	2 (10.5)
cornea verticillata, n	0 (0.0)	0 (0.0)	0 (0.0)	9 (19.6)	8 (29.6)*	1 (5.3)
LVEF, %	63.1 ± 6.4	60.9 ± 6.2	66.2 ± 5.6	63.2 ± 9.6	64.5 ± 9.8	60.6 ± 8.8

Values are given as mean ± SD for continuous data or n (%) for categorical data. Continuous values were compared using unpaired Student’s t test and categorical values have been tested with Fisher’s exact test (both two-sided). LVEF: left ventricular ejection fraction. Patients have been gender-specific compared. *p < 0.05, **p < 0.01

**Table 2:** Clinical data and parameters

	p.A143T patients			Missense mutation patients		
	Total (*n* = 25)	Female (*n* = 15)	Male (*n* = 10)	Total (*n* = 58)	Female (*n* = 39)	Male (*n* = 19)
age, years	46.3 ± 13.7	48.7 ± 14.3	42.7 ± 12.6	44.1 ± 16.6	47.7 ± 17.3	36.8 ± 12.6
body weight, kg	76.6 ± 20.4	65.8 ± 10.7	92.7 ± 21.4	75.8 ± 19.6	71.6 ± 19.1	83.8 ± 18.3
body height, cm	172.0 ± 10.9	166.1 ± 5.1	181.0 ± 11.3	171.9 ± 9.4	167.0 ± 5.9	181.6 ± 7.2
BMI, kg/m^2^	25.6 ± 4.8	24.0 ± 4.3	28.0 ± 4.5	25.5 ± 5.8	25.6 ± 6.3	25.3 ± 4.8
GLA activity (normal range 100–250 %) , % of reference	65 [25–181]	100 [54–181]	48 [25–72]	80 [0–272]	166 [34–272]*	13 [0–69]**
lyso-Gb3 within reference range (<0.9 ng/ml), n	19 (82.6)	12 (85.7)	7 (77.8)	9 (22.5)	8 (30.8)**	1 (7.1)***
lyso-Gb3, ng/ml	0.7 ± 0.3	0.6 ± 0.3	0.8 ± 0.3	13.0 ± 16.0	4.5 ± 4.8**	30.2 ± 16.9^****^
ERT, n^a^	10 (40.0)	4 (26.7)	6 (60.0)	25 (43.1)	12 (30.8)	13 (68.4)
Fabry crisis, n	2 (8.0)	2 (13.3)	0 (0.0)	8 (15.1)	5 (14.7)	3 (15.8)
stroke/TIA, n	7 (28.0)	3 (20.0)	4 (40.0)	5 (8.6)	2 (5.1)	3 (15.8)
IVSd, mm	9.0 ± 1.8	8.3 ± 1.8	9.8 ± 1.4	11.6 ± 5.0	10.6 ± 3.2**	14.0 ± 7.3*
LVH (IVSd > 12 mm), n	3 (12.0)	1 (6.7)	2 (20.0)	19 (40.4)	11 (33.3)	8 (57.1)
pacemaker, n	0 (0.0)	0 (0.0)	0 (0.0)	1 (1.7)	1 (2.6)	0 (0.0)
NYHA class, n						
0	8 (33.3)	3 (21.4)	5 (50.0)	9 (15.5)	7 (18.0)	2 (10.5)*
I	15 (62.5)	10 (71.4)	5 (50.0)	36 (62.1)	23 (59.0)	13 (68.4)
II	1 (4.2)	1 (7.1)	0 (0.0)	10 (17.2)	8 (20.5)	2 (10.5)
III	0 (0.0)	0 (0.0)	0 (0.0)	3 (5.2)	1 (2.6)	2 (10.5)
IV	0 (0.0)	0 (0.0)	0 (0.0)	0 (0.0)	0 (0.0)	0 (0.0)
ACR, mg albumin/ g creatinine	7 [0–14323]	12 [0–14323]	1 [0–12]	82 [0–4080]	59 [0–4080]***	195 [0–2668]****
albuminuria (>30 mg/g), n	4 (16.7)	4 (28.6)	0 (0.0)	37 (82.2)	22 (75.9)**	15 (93.8)****
creatinine, mg/dl	0.86 ± 0.31	0.80 ± 0.35	0.96 ± 0.20	0.86 ± 0.38	0.73 ± 0.18	1.12 ± 0.53
eGFR, ml/min/1.73 m^2^	95.6 ± 17.6	95.0 ± 16.5	96.4 ± 20.1	97.3 ± 27.5	98.3 ± 21.4	95.2 ± 37.6
dialysis, n	0 (0.0)	0 (0.0)	0 (0.0)	1 (1.7)	0 (0.0)	1 (5.3)
NTX, n	0 (0.0)	0 (0.0)	0 (0.0)	1 (1.7)	0 (0.0)	1 (5.3)
CKD stage, n						
G1 (≥90 ml/min/1.73 m^2^)	18 (72.0)	11 (73.3)	7 (70.0)	37 (67.3)	26 (70.3)	11 (61.1)
G2 (60–89 ml/min/1.73 m^2^)	6 (24.0)	4 (26.7)	2 (20.0)	12 (21.8)	9 (24.3)	3 (16.7)
G3 (30–59 ml/min/1.73 m^2^)	1 (4.0)	0 (0.0)	1 (10.0)	6 (10.9)	2 (5.4)	4 (22.2)
G4 (15–29 ml/min/1.73 m^2^)	0 (0.0)	0 (0.0)	0 (0.0)	0 (0.0)	0 (0.0)	0 (0.0)
G5 (<15 ml/min/1.73 m^2^)	0 (0.0)	0 (0.0)	0 (0.0)	0 (0.0)	0 (0.0)	0 (0.0)
RAAS blockers, n	8 (32.0)	5 (33.3)	3 (30.0)	18 (54.6)	10 (43.5)	8 (80.0)*
diuretics, n	2 (8.0)	0 (0.0)	2 (20.0)	5 (16.1)	5 (23.8)	0 (0.0)
analgesics, n	2 (8.0)	1 (6.7)	1 (10.0)	6 (21.4)	6 (30.0)	0 (0.0)
antidepressants, n	3 (12.0)	2 (13.3)	1 (10.0)	4 (14.8)	4 (21.1)	0 (0.0)
MSSI score (max. 76)	8.5 ± 7.8	8.1 ± 6.7	9.0 ± 9.7	8.9 ± 6.7	8.1 ± 7.5	10.4 ± 4.7
MSSI general (max. 18)	2.4 ± 1.9	2.8 ± 2.3	1.8 ± 1.2	1.8 ± 1.6	1.4 ± 1.6*	2.4 ± 1.3
MSSI cardiac (max. 20)	0.9 ± 2.5	0.6 ± 2.4	1.2 ± 2.6	2.7 ± 4.2	2.9 ± 4.1*	2.3 ± 4.4
MSSI renal (max. 18)	2.1 ± 3.6	2.3 ± 3.8	1.8 ± 3.5	2.4 ± 2.8	1.8 ± 2.8	3.6 ± 2.6*
MSSI neurologic (max. 20)	3.1 ± 4.1	2.4 ± 3.7	4.1 ± 4.6	2.1 ± 2.2	2.1 ± 2.3	2.2 ± 2.2
total DS3 score (max. 80)	6.8 ± 6.5	6.3 ± 6.3	7.6 ± 7.1	9.0 ± 6.2	7.4 ± 6.0	12.1 ± 5.6
total DS3 cardiac (max. 24)	0.6 ± 1.8	0.4 ± 1.3	1.0 ± 2.3	2.8 ± 3.3	2.6 ± 3.2*	3.3 ± 3.5
total DS3 renal (max. 24)	1.3 ± 2.1	1.6 ± 2.3	1.0 ± 1.7	2.4 ± 3.4	1.9 ± 3.0	3.6 ± 3.9*
total DS3 neurologic (max. 16)	4.4 ± 4.7	3.9 ± 4.4	5.2 ± 5.3	3.4 ± 3.5	2.8 ± 3.3	4.8 ± 3.7

Missense mutations were restricted to amino acid substitutions due to single nucleotide mutations within the coding region absent of the catalytic active protein sites. Values are given as mean ± SD, median [range] for continuous data or n (%) for categorical data. Continuous values were compared using unpaired Student’s t test or Mann Whitney U test if unequal distribution was observed (both two-sided). Categorical values have been tested with Fisher’s exact test (two-sided). Reference values for plasma lyso-Gb3 < 0.9 ng/ml and GLA activity >32 nmol/h/mg protein in leukocytes. To compare GLA activities between laboratories, values are presented as the percentage of the laboratory reference value. ACR: albumin-to-creatinine ratio; BMI: body mass index; CKD: chronic kidney disease; DS3: total Disease Severity Scoring System; eGFR: estimated glomerular filtration rate (calculated via CKD-EPI formula); ERT: enzyme replacement therapy; IVSd: interventricular septum thickness in diastole; LVH: left ventricular hypertrophy; MSSI: Mainz Severity Score Index; NTX: renal transplantation; NYHA: New York Heart Association; RAAS: renin-angiotensin-aldosterone-system. TIA: transient ischemic attack. ^a^ERT initialization within 12 months after 1.^st^ visit. *p < 0.05, **p < 0.01, ***p < 0.001, ****p < 0.0001

Mean plasma lyso-Gb3 levels in p.A143T patients were significantly lower than in patients with classical missense mutations, so that most p.A143T patients showed normal lyso-Gb3 values (lyso-Gb3 < 0.9 ng/ml: females: 85.7 %; males: 77.8 %, Table 2). Linear regression analysis revealed that male FD patients with missense mutations showed decreased GLA activities, which were significantly associated with increased lyso-Gb3 values (*p* = 0.0017; r^2^ = 0.6410; Fig. 2b). In this analysis a residual GLA activity of <40–50 % was always associated with increased lyso-Gb3 levels (Fig. 2b). In contrast, in male p.A143T patients such a correlation was statistically not significant (*p* = 0.2103), since most males showed only a slightly decreased GLA activity with a normal lyso-Gb3 level (Fig. 2c).

In addition, p.A143T patients (females as well as males) had lower values for IVSd (*p* < 0.01 and *p* < 0.05, respectively; Table 2), resulting in lower frequencies of LVH. This mild cardiac involvement is also reflected by the distribution of the New York Heart Association (NYHA) classes, as half of the male p.A143T patients were classified in NYHA class 0, whereas ~90 % of the typical FD patients suffered from a more severe heart failure (NYHA class II-IV, Table 2). The PR intervals seen on *ECG* in p.A143T patients were also in a normal range (mean: 157 ± 20 ms) and not shortened as typical for FD [24]. All p.A143T patients had a lower ACR (*p* < 0.001) compared to other FD missense mutation patients, resulting in a lower risk for albuminuria (females: *p* = 0.0031, males: *p* < 0.0001; Table 2). The eGFR values and raw serum creatinine levels were comparable in all analyzed patients (Table 2). Male patients with missense mutation were more often treated with RAAS blockers (Table 2). The mild organ involvement was also demonstrated by the evaluation of disease scores in that patients with p.A143T (females as well as males) presented with lower values of MSSI and DS3 subscores as patients with other missense mutations (Table 2). In detail, female p.A143T patients had lower cardiac scores (both, MSSI and DS3) and males lower renal scores (both, MSSI and DS3). Furthermore, in contrast to patients with other missense mutations (MSSI: *p* = 0.0076), increasing MSSI scores were not associated with increasing age (MSSI: *p* = 0.4593).

According to the TOAST criteria [17] six additional (2 heterozygous females and 4 hemizygous males) unrelated p.A143T patients with a mean age of 41.3 ± 5.6 years (age at event) suffered from stroke (*n* = 5) or TIA (*n* = 1) of either unknown (*n* = 4), macroangiopathic (*n* = 1), or other origin (A. carotis interna dissection, *n* = 1) before their first assessment. Of note, similar to the index patient, four patients with cryptogenic stroke/TIA were the first of their families presenting at the participating FD centers. Although subsequent family screening revealed more p.A143T relatives, none of these suffered from stroke/TIA. Additionally, family members without p.A143T were free of neurological events.

Figure 1 shows representative magnet resonance images (MRI) of three p.A143T patients (1 male/ 2 females) suffering from stroke/TIA. Both females showed random X chromosome inactivation (allele ratios for index patient 50:50 and TIA patient: 60:40) in blood samples (leukocytes) (Additional file 2: Figure S1).

Of note, p.A143T patients suffering from stroke/TIA had slightly decreased residual GLA activities, and/or increased lyso-Gb3 levels.

## Discussion

To determine the clinical impact of the *GLA* p.A143T variation, we retrospectively analyzed 25 heterozygous (females) and hemizygous (males) adult Fabry patients with this variation and compared gender-specific their clinical symptoms and manifestations with a well-characterized group of 58 FD patients (39 heterozygous females) with other classical missense mutations.

Our main findings are: 1) Most male p.A143T patients had only slightly decreased residual GLA activities and normal lyso-Gb3 levels, while GLA activity in heterozygous females was normal; 2) Female and male p.A143T patients showed a less organ involvement in comparison to FD patients with other missense mutations; 3) Female and male p.A143T patients suffering from stroke/TIA showed no further FD-typical organ manifestations; 4) No accumulation of neurologic events in family members of p.A143T patients with stroke/TIA was observed.

Recent studies suggested the p.A143T variation to be more likely a neutral variant or a possible modifier than a disease causing mutation [10, 13]. In our study, the risk for FD-typical manifestations such as albuminuria, increased IVSd, and increased disease severity scores was significantly lower in male as well as female p.A143T patients when compared to groups with classical FD missense mutations. The higher disease burden of FD patients with other missense mutations is also demonstrated by severely decreased GLA activities and/or increased lyso-Gb3 levels, while most p.A143T patients (females and males) showed normal values. However, the residual enzymatic activities in male p.A143T patients revealed a bright variance between 25 to 72 % of normal GLA activity, which has also been observed by other investigators especially in cultured T cells [24]. The only moderately decreased GLA activity in leukocytes of p.A143T males seems to be sufficient to keep plasma lyso-Gb3 levels below the reference value. The ultimate presence or absence of cellular Gb3 deposits can be diagnosed by appropriate tissue biopsies (“gold standard”), which have not been performed in our study. However, Terryn and colleagues performed cardiac biopsy in one female patient with p.A143T with LVH and kidney biopsies in two p.A143T males with renal failure and microalbuminuria, demonstrating no Gb3 deposits [13]. Hence, the authors concluded that the presence of this mutation is not directly associated with FD-pathology (i.e. Gb3 deposits) and should not be labeled as “pathogenic” [13]. The authors also stated that the lack of Gb3 deposits does not preclude elevated plasma lyso-Gb3 levels that could be pathogenic and cause endothelial cell dysfunction, and suggested that this should be analyzed in further studies [13]. Similar to recent studies [9, 10, 13] the risk for patients with p.A143T in our study with only slightly reduced GLA activities (males) and/or normal lyso-Gb3 levels (males and females) for renal and cardiac end organ damage was reduced in comparison to other FD patients with classical missense mutations. In addition, instead of a shortened PR interval on ECG, which would be typical for a storage disease [25, 26], the PR interval in our p.A143T patients was normal, demonstrating also no FD-typical cardiac involvement. The lack of FD-typical manifestations might be also reflected by the absence of an age effect on the MSSI score within the p.A143T patients.

Including the index patient, we identified at least 5 neurologic events (stroke or TIA) in heterozygous (females) as well as hemizygous (males) patients with p.A143T of unknown etiology, which might be assigned to FD. This would be in line with recent observations suggesting that p.A143T may be associated with a "stroke-only" phenotype [8, 27]. Due to the fact that the neurologic system seems to be highly sensitive to reduced GLA activity with neuropathic pain as one of the first symptoms and early neurologic manifestations such as stroke and TIA [28, 29], the slightly reduced GLA activities observed in p.A143T patients might predominantly manifest in the central nervous system. However, strokes in FD look like strokes from other causes and the high frequency of neurological events in our p.A143T patients (20.0 %) in comparison to missense mutation patients (8.6 %) seems to be based on a screening bias. According to the Exome Aggregation Consortium (ExAC, http://exac.broadinstitute.org/) the population frequency of c.427G > A is 0.078 % in the European population, demonstrating a not infrequent finding of this variation. If the p.A143T variation alone would be pathogenic, significant more patients with even this variation with FD-typical signs and symptoms should have been identified by the participating FD centers. Our GLA expression analysis revealed no decreased mRNA expression compared to healthy controls, suggesting that p.A143T seems to be more likely a neutral variant. However, it might be possible, that the variant acts as a modifier of a yet unknown causal factor.

## Conclusions

With respect to our current findings and in accordance with previous reports, which demonstrate the lack of Gb3 deposits within affected organs [13], we conclude that p.A143T seems not to be causal for FD but rather a genetic variant of unknown significance or a genetic modifier. Our data confirm previous findings that lyso-Gb3 levels seem to be mostly normal in atypical FD patients [30] or patients with genetics variants of unknown significance [10].

We suggest that p.A143T patients with stroke/ TIA of unknown etiology should be further evaluated, since the diagnosis of FD is not probable and ERT or chaperone treatment should not be an unreflected option.

### Limitations

Possible restrictions of this study are the retrospective design and the limited number of patients. Due to the multicenter approach, some parameters (such as lyso-Gb3 values) and DNA samples for X-chromosome inactivation analysis were not available for the entire study cohort. Since DNA for the two analyzed females was only available from leukocytes, according to Echevarria and colleagues a skewed X chromosome inactivation within other tissues cannot be excluded [31]. The lack of tissue biopsies demonstrating the ultimately presence or absence of cellular Gb3 deposits is a limitation. A clear link between genotype and phenotype in FD is difficult to be established especially in young patients presented with stroke since it is usually not possible to document GB3 deposit in the central nervous system.

## Additional files

Additional file 1: Table S1. Overview of different missense *GLA* mutations (*n* = 24) within the study cohort served as controls. (DOC 57 kb) Additional file 2: Figure S1. X chromosome inactivation analysis in two female p.A143T patients suffering from stroke and TIA. (DOC 3993 kb)

## Abbreviations

ACR: albumin/creatinine ratio
BMI: body mass index
CI: confidence interval
CKD: chronic kidney disease
DS3: Disease severity scoring system
ECG: electrocardiogram
eGFR: estimated glomerular filtration rate
ERT: enzyme replacement therapy
FD: Fabry disease
Gb3: globotriaosylceramide
GLA: alpha-galactosidase A
IVSd: inter ventricular systolic diameter
LVH: left ventricular hypertrophy
MSSI: Mainz Severity Score Index
NTX: renal transplantation
NYHA: New York Heart Association
OR: odds ratio
PCR: polymerase chain reaction
RAAS: renin-angiotensin-aldosterone-system
TIA: transient ischemic attack
TOAST: Trial of ORG 10172 in Acute Stroke Treatment
