# Supplementary material for: Alpha-Galactosidase A p.A143T, a non-Fabry disease-causing variant
Source: Orphanet J Rare Dis. 2016 May 4;11:54. doi: 10.1186/s13023-016-0441-z (PMC4855861; doi:10.1186/s13023-016-0441-z)
Supplement: Additional file 1: Table S1. — Overview of different missense GLA mutations (n = 24) within the study cohort served as controls. (DOC 57 kb) [file 13023_2016_441_MOESM1_ESM.doc]

| **Table S1: Overview of different missense *GLA* mutations (n=24) within the study cohort served as controls.** | | | | | |
| --- | --- | --- | --- | --- | --- |
| **Missense mutations** | | | | | |
| **Amino acid** | **Clinical phenotype** | **actual mean lyso-Gb3*, ng/ml** | **Reference No.** | **comparable mutation or protein damage** | **Accession number** |
| **p.A20P** | mild (female) | 3.3 | Nakao (1995) N Engl J Med 333, 288 |  | CM950584 |
| **p.G35E** | classic | 23.4 | **own data** | p.G35R; Davies (1994) Hum Mol Genet 3, 667 |  |
| **p.L45P** | classic | 31.6 | **own data** | disruption of beta sheet at AS position L45 |  |
| **p.I91T** | classic | 1.1 | Eng (1997) Mol Med 3, 174 |  | CM972767 |
| **p.C94S** | classic | 1.7 | Blaydon (2001) Hum Mutat 18, 459 |  | CM012955 |
| **p.R112C** | mild | nd | Ishii (1992) Hum Genet 89, 29 |  | CM920311 |
| **p.R118C** | mild | 0.7 | Spada (2006) Am J Hum Genet 79, 31 |  | CM061789 |
| **p.A121T** | mild (female) | 0.4 | **own data** | p.A121P; Lorenz (2003) Wien Klin Wochenschr 115, 235 |  |
| **p.W162G** | classic | 24.5 | Lukas (2013) PLOS Genetics 9, e1003632 |  |  |
| **p.W162C** | classic | 15.5 | Germain (1996) Hum Genet 98, 719 |  | CM960766 |
| **p.V164G** | classic | 19.5 | **own data** | no direct secondary structure disruptions |  |
| **p.G183S** | classic | nd | Shabbeer (2002) Mol Genet Metab 76, 23 |  | CM023791 |
| **p.M187V** | classic | 11.4 | Ashton-Prolla (2000) J Investig Med 48, 227 |  | CM003747 |
| **p.K213M** | classic | 5.6 | Lukas (2013) PLOS Genetics 9, e1003632 |  |  |
| **p.I242V** | mild (female) | 0.5 | **own data** | p.I242N; Takata (1997) Brain Dev 19, 111 |  |
| **p.R252T** | classic | 0.5 | Lukas (2013) PLOS Genetics 9, e1003632 |  |  |
| **p.P259R** | classic | 44.8 | Ashley (2001) J Hum Genet 46, 192 |  | CM012375 |
| **p.M267T** | classic | 0.8 | Lukas (2013) PLOS Genetics 9, e1003632 |  |  |
| **p.L294S** | classic | 26.1 | Lukas (2013) PLOS Genetics 9, e1003632 |  |  |
| **p.R301P** | classic | 14.7 | Ashley (2001) J Hum Genet 46, 192 |  | CM012377 |
| **p.R301Q** | classic | 14.9 | Sakuraba (1990) Am J Hum Genet 47, 784 |  | CM900111 |
| **p.I319T** | classic | 3,5 | Sirrs (2010) Mol Genet Metab 99, 367 |  | CM101631 |
| **p.G325S** | classic | 6.4 | Schafer (2005) Hum Mutat 25, 412 |  | CM051536 |
| **p.G328R** | classic | 17.7 | Ishii (1992) Hum Genet 89, 29 |  | CM920312 |
| *under enzyme replacement therapy. | | | | | |
