# Supplementary material for: Alpha-Galactosidase A p.A143T, a non-Fabry disease-causing variant
Source: Orphanet J Rare Dis. 2016 May 4;11:54. doi: 10.1186/s13023-016-0441-z (PMC4855861; doi:10.1186/s13023-016-0441-z)
Supplement: Additional file 2: Figure S1. — X chromosome inactivation analysis in two female p.A143T patients suffering from stroke and TIA. (DOC 3993 kb) [file 13023_2016_441_MOESM2_ESM.doc]

| 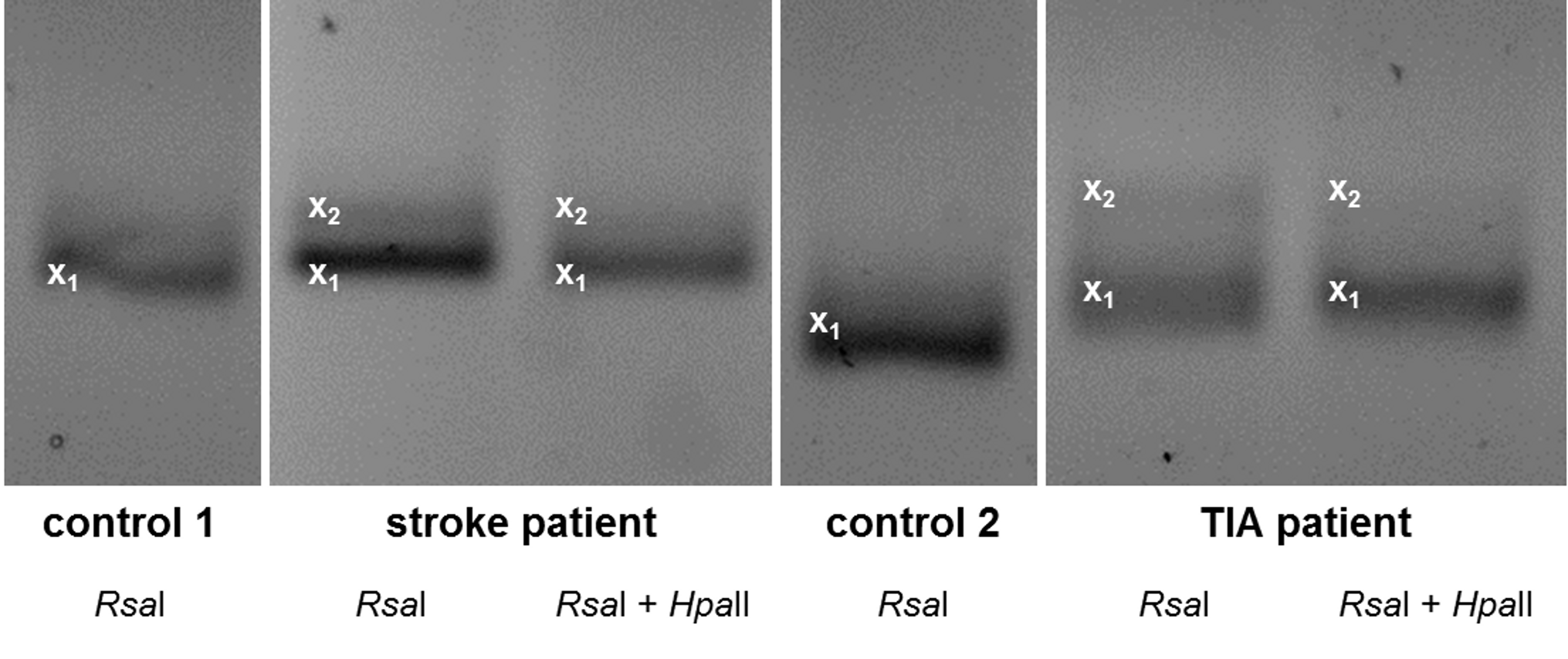 |
| --- |
| **Figure S1: X chromosome inactivation analysis in two female p.A143T patients suffering from stroke and TIA.** Control 1: p.143T allele of the stroke patient’s father. Control 2: p.143T allele of the TIA patient’s son. X_1_: p.143T allele. X_2_: healthy p.A143 allele. |
